# Maxillary first molar with three mesiobuccal canals confirmed with spiral computer tomography

**DOI:** 10.4317/jced.50850

**Published:** 2012-10-01

**Authors:** Pooja Kakkar, Anant Singh

**Affiliations:** 1 MDS, Professor and Head. Department Of Conservative Dentistry and Endodontics. Sardar Patel Post Graduate Institute of Dental And Medical Sciences, Lucknow.; 2 Post Graduate. Department Of Conservative Dentistry and Endodontics. Sardar Patel Post Graduate Institute of Dental And Medical Sciences, Lucknow.

## Abstract

Anatomic variations in root canal morphology have become easier to detect with the rapid advancements in clinical and diagnostic aids. Also an increased awareness of unusual anatomic morphology and a better understanding of the root canal system guides the clinician in diagnosis and treatment of such variations in order to achieve a successful endodontic outcome. Mesiobuccal root of first maxillary molar teeth have been considered to be one of the most complex and challenging root canal systems. This case report presents a successful management of a third canal in the mesiobuccal root of permanent maxillary first molar using dental operating microscope along with a modified access preparation and confirmed with the aid of spiral computed tomography.

** Key words:**Maxillary first Molar, Three mesiobuccal canals, Spiral CT, Anatomic variation.

## Introduction

Permanent first molar teeth are one of the first teeth to erupt into the oral cavity and therefore possibly the most root canal indicated tooth for the endodontist.

The most common cause of treatment failures in permanent maxillary first molars have been attributed to failure in detecting additional canals especially in the mesiobuccal root and therefore has resulted in more research and clinical investigation than any other root.

In 1969 Weine et al (1) provided the first clinical classification of more than one canal system in a single root and used the mesiobuccal root of the maxillary first molar as the type specimen.

Studies specifically addressing the mesiobuccal root have reported that the incidence of extra root canals in vitro is greater than in vivo. Apart from this, a wide variation of root and canal configurations of the maxillary first molars have been documented in the dental literature. Most of the in vitro studies addressing the mesiobuccal root canal anatomy (2,3) have not reported the presence of a third canal in the mesiobuccal root. Two such studies have reported their incidence to be between 1.1% and 10% (4,5). However its presence has been documented in only a few case reports (6,7).

A case report is presented that illustrates multiple root canal systems that occur in the mesiobuccal root of maxi-llary first molar and its successful nonsurgical root canal treatment using modified access cavity preparation. This unusual morphology was confirmed with the aid of spiral computed tomography (CT) scanning.

## Case Report

A Nineteen year old male patient of Indian descent was referred to the Post Graduate Department of Conservative Dentistry and Endodontics with the chief complaint of intermittent pain over three months in relation to the upper right maxillary first molar region. The patient also complained of episodes of sensitivity to hot foods in the involved tooth. Medical and dental history was non-contributory.

On clinical examination, patient’s oral hygiene was found to be moderate. Deep occlusal carious lesion was observed in maxillary right first molar and was tender on percussion. Electric pulp test (Sybron Endo, Orange, CA,USA) and heat test with gutta percha stick gave a lingering response. There was no evidence of swelling or sinus tract.

**Figure 1:**
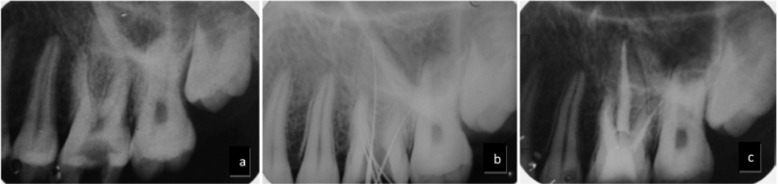
IOPA radiographs of the maxillary first molar. a. Pre-operative radiographs; b. Shows the working length radiograph ; c. Post-obturation radiograph.

Preoperative periapical radiographic examination revealed widening of lamina dura (Fig. 1). Based on clinical and radiographic evidences a diagnosis of irreversible pulpitis was made and the tooth was prepared for nonsur-gical endodontic treatment.

Access was gained into the pulp chamber using after administration of local anesthesia, 2% Lignocaine with 1:80,000 adrenaline (Septodont, Cedex, France) under rubber dam isolation.

Initially, the mesiobuccal, distobuccal and palatal canals were located. On the floor of the pulp chamber near the mesiobuccal canal, a soft area was detected which was then carefully probed using a DG-16 endodontic explorer (Hu Freidy ,Chicago, IL, USA). Upon probing this area, two additional bleeding points were clinically observed. Assuming these bleeding points to be additional canals in the mesiobuccal root, pathfinder files (Sybron Endo, Glendora, CA,USA) were introduced as exploratory files and were negotiated to a considerable distance with relative ease.

The conventional triangular access was modified to a rhomboidal shape to improve access to the additional canals The three canal orifices in the mesiobuccal root were located under magnification in using dental operating microscope (Fig. 2) (Zeiss, Oberkochen, Germany).

**Figure 2:**
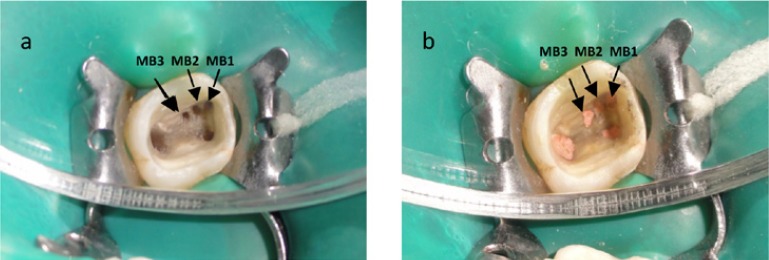
2. a. Photographs showing five canals after access opening ; b. Post obturation photograph.

The radiographical working length was confirmed with an electronic apex locator (Sybron Endo, Orange, CA, USA). A radiograph was taken which confirmed the presence of three canals in the mesiobuccal root (Fig. 1).

Rubber dam clamp was removed while taking radiograph between the procedure so as to get a clear image of three separated canals in the MB root at the cervical third of the root.

Nickel titanium protaper series orifice shapers (Dentsply, Maillifer, Ballaigues, Switzerland) was used to enlarge the orifice to achieve a straight line access to the apex. The canals were cleaned and shaped sequentially with rotary Protaper files (Dentsply, Maillifer, Ballaigues, Switzerland). During preparation EDTA (Glyde File Prep. Dentsply, Maillifer, Ballaigues, Switzerland) was used as lubricant and root canal were disinfected with 3% sodium hypochlorite (Prevest Denpro Ltd. Digiana, Jammu, India) followed by a final rinse with saline and dried with paper points.

The palatal and distobuccal canals were obturated with F3 and F2 protaper gutta percha cones respectively (Dentsply, Maillifer, Ballaigues, Switzerland). The mesiobuccal canal (MB-1) was obturated with F2 protaper gutta percha cones (Dentsply, Maillifer, Ballaigues, Switzerland). MB-2 and MB-3 were obturated with F1 pro-taper gutta percha cones (Dentsply, Maillifer, Ballaigues, Switzerland). AH- plus (Dentsply, Destrey, Konstanz, Germany) was used as the sealer. ( Fig. 2).

All three canals in the mesiobuccal root could not be visualized separately after obturation because of the two dimensional nature of the radiographs (Fig. 1).

**Figure 3:**
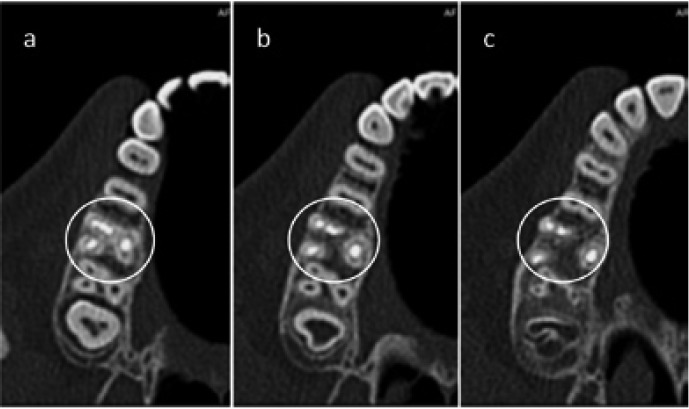
Spiral CT images; a.Cervical third; b.Middle third; c.apical third.

A spiral CT scan (Siemens, Munich, Germany) was performed at this point after taking an informed consent from the patient. Transverse slices of the molar were obtained at different levels to determine the canal morphology. CT slices of the Dentascan revealed three mesiobuccal canals with three separate orifices and two apices with a very thin dentinal separation between MB-2 and MB-3 canals (Fig. 3).

The access cavity was filled with GIC (Chem Flex, Dentsply, Destrey, Konstanz, Germany) and patient was kept on recall for fixed prosthesis.

## Discussion

Traditionally, many treatment failures in maxillary molars were related to not locating additional canals in the mesiobuccal root.

Despite the current high success rate achieved by root canal therapy, the permanent maxillary first molars is still associated with a considerable number of failures mainly in the mesiobuccal root due to the difficulty in locating and filling the second and/or third mesiobuccal canals (8,9). The causes of missed canals could be attributed to the limited knowledge of the root canal system, incomplete access preparations and clinician’s dependency on two dimensional radiographs, to name a few.

These obstacles can be overcome by updating our knowledge on the complexities of the root canal system and realizing the importance of modified access preparations in such cases so as to facilitate in the detection and location of additional canals.

The use of conventional radiographs for interpretation of complex root canal anatomy in the mesiobuccal roots of maxillary first molar could fall short due to superimpostion of maxillary sinus, zygomatic buttress or overlapping between the roots. Detection of a third mesiobuccal root canal could be hindered radiographically because the locations of additional canals in the mesio buccal root occur on the same plane. Hence, additional periapical radiographs should be taken by varying the horizontal angle followed by detailed examination of the radiographs.

The working length radiograph is the most informative radiograph for locating extra canals. If the endodontic files are not well centered in the canal on the radiograph, the possibility of additional canals should always be considered. Also the extra mesiobuccal canal will often show up as a dark line running nearly parallel to the file in the coronal third of the root (10).

The establishment of adequate access to the entire pulp chamber is the most important step in successfully locating the additional canals in the mesiobuccal root.

A number of investigators concluded that the preparation of a rhomboidal shaped access cavity rather than a traditional triangular outline form would permit straight line visualization, allowing for complete debridement of the pulp chamber and aid in localization of additional canals in the mesiobuccal root of the maxillary molars.

In approximately 30% of cases, the additional use of probing with sharp explorers or cutting instruments such as a round slow speed bur to trough along the subpulpal groove starting from the main mesiobuccal canal within the pulp chamber floor is necessary to locate any additional canals in this root (11).

In this case, the third canal was located by modifying the access cavity from the traditional triangular outline form to a rhomboidal shape which permitted straight line visualization, allowing for complete debridement of the pulp chamber and aided in localization of the MB 3 canal in the mesiobuccal root of the maxillary first molar. The young age of the patient involved in this case may have contributed to the relative ease of identification of the third canal without the use of a SOM. This fact corroborates the findings of Iqbal and Filmore who found a correlation between caries, treatment regimens and patient age in the ultimate detection of the number of canals by the clinician in maxillary molars (12).

In this case, the modified access preparation allowed for a clear view of the pulp chamber floor that showed the presence of three bleeding points in the mesiobuccal root. Other diagnostic aids for canal location are staining the pulpal floor with opthalmic dyes, transillumination or performing the champagne or bubble test with warmed 2.6% Naocl as suggested by Ruddle (13).

Studies with modern techniques supported by magnification and adequate illumination have reported higher rates of detection of a second canal in the mesiobuccal root of maxillary molars (14). Even so, more emphasis should be placed on the importance of using magnification in locating MB 3 canal and not on which type of magnification is used. In our case SOM helped in complete negotiation and root canal therapy of all three canal successfully.

Spiral CT was taken after obturation which confirmed the presence of three canals in the mesiobuccal root as mesiobuccal-1,2 and 3 (MB1 MB2 MB3). In the case reported in this paper, the mesiobuccal root appeared to present minimal root curvature with three individual orifices on the pulpal floor. MB-1,showed one opening and one apical exit (Vertucci’s first class), while MB-2 and MB-3 presented with two separate openings and one apical exit (Vertucci’s class four) (Fig. 3)

To conclude, the advent of 3D imaging has provided the endodontist with sophisticated diagnostic tools for effective evaluation of root canal morphology that were not available to the clinician before and facilitated interactive image manipulation and enhancement to visualize the area of interest (15). However in general its high cost, accessibility and availability to patient and extra radiation as compared to standard radiographic methods makes its routine use limited especially in a third world country setting. Therefore a thorough knowledge of root canal anatomy and its variations, careful interpretation of the radiographs, close clinical inspection of the floor of the chamber and proper modification of access opening along with adequate magnification are essential for successful treatment outcome.
